# Good Veterinary Practices for Managing the Colostral Period in Dairy Calves to Improve Their Immune Competence—A Systematic Review

**DOI:** 10.3390/vetsci12121205

**Published:** 2025-12-16

**Authors:** Elena Stancheva, Toncho Penev

**Affiliations:** Department of Ecology and Animal Hygiene, Faculty of Agriculture, Trakia University, 6000 Stara Zagora, Bulgaria; tonchopenev@abv.bg

**Keywords:** colostrum, IgG, passive transfer, newborn calves, good veterinary practices

## Abstract

Passive transfer of immunoglobulin G (IgG) through colostrum is essential for early immune protection in dairy calves. This systematic review synthesizes the most relevant evidence on colostrum quality, feeding practices, storage methods, and post-feeding assessment. High-quality colostrum (≥50 g/L IgG and low bacterial contamination) can be reliably assessed using Brix refractometry (≥22%) or radial immunodiffusion. Early administration is critical: feeding within 1–2 h after birth maximizes IgG absorption. Providing 8.5–10% of body weight (≈3–4 L) at first feeding, followed by a second feeding within 8–12 h, significantly reduces failure of passive transfer. Pasteurization at 60 °C for 60 min and controlled freezing maintain immunological integrity while reducing microbial load. Monitoring passive immunity using serum total protein (5.0–5.5 g/dL) or serum Brix (8.1–8.5%) offers practical field diagnostics aligned with the IgG ≥ 10 g/L threshold. Integrated colostrum management protocols—combining quality evaluation, timely feeding, hygienic handling, and trained personnel—are essential to improve passive immunity, health, and overall performance of dairy calves.

## Simple Summary

Passive transfer of immunity through colostrum is crucial for the health and productivity of neonatal calves. Colostrum quality, timing and volume of the first feeding, feeding frequency, and proper storage determine the effectiveness of this process. Well-structured protocols, supported by good veterinary practices and trained personnel, significantly reduce the risk of failure of passive transfer and improve calf health. Integrated management of the colostral period is essential for sustainable and efficient dairy production.

## 1. Introduction

Passive transfer of immunity (PTI) achieved through the timely intake of high-quality colostrum remains one of the central determinants of neonatal calf health, survival and later productivity in dairy herds [1]. Since the synepitheliochorial placenta of the cow prevents the transplacental transfer of immunoglobulins, calves are born agammaglobulinemic and depend entirely on colostrum-derived antibodies to establish systemic immunity [2]. Early ingestion and efficient intestinal absorption of immunoglobulin-rich colostrum are therefore essential for reducing the risk of disease and mortality in the first weeks of life [3].

The “Three Qs” concept—Quality, Quantity, Quickly—has been widely adopted as a guiding framework for colostrum management. It emphasizes that newborn calves should receive colostrum of adequate IgG concentration, in sufficient volume and as soon as possible after birth [4,5]. This framework is supported by classical studies describing the kinetics of immunoglobulin absorption and the rapid decline in intestinal permeability during the hours following parturition [6,7].

Despite the widespread dissemination of these principles, substantial variation in colostrum quality and management practices continues to be reported across dairy farms. A large nationwide survey in the United States, for example, found wide variation in colostrum IgG concentrations, with a considerable number of samples falling below recommended thresholds [8]. Recent publications similarly highlight that inadequate passive transfer remains common, underlining the need for standardized, evidence-based protocols for colostrum collection, handling and administration [9].

Maternal characteristics contribute significantly to the variation observed in colostrum composition. Multiparous cows generally produce colostrum with higher IgG concentrations than primiparous animals [10], which supports the practice of maintaining colostrum banks based on frozen colostrum from healthy older cows.

An invited review by Westhoff et al. [11] provides a detailed overview of current knowledge regarding nutritional and management factors affecting colostrum production and composition. However, the review offers limited guidance on practical on-farm protocols for evaluating and managing colostrum quality. This gap underscores the need for a systematic review focused specifically on practical, evidence-based strategies aimed at optimizing colostrum management and improving passive immunity in dairy calves.

The aim of this systematic review is to:

- Summarize current evidence on methods for assessing colostrum quality, including laboratory-based and on-farm rapid techniques;
- Analyze management practices related to collection, hygiene, storage, processing, and delivery of colostrum;
- Evaluate factors influencing immunoglobulin absorption and determinants of successful passive transfer;
- Provide practical, evidence-based recommendations for improving newborn calf immunity through optimized colostrum management.

## 2. Materials and Methods

### 2.1. Study Design

This review was performed in accordance with the PRISMA (Preferred Reporting Items for Systematic Reviews and Meta-Analyses) guidelines. The review aimed to synthesize evidence on colostrum quality assessment, colostrum management practices, and indicators used for evaluating PTI in dairy calves. Only peer-reviewed scientific publications reporting original data were included. The review protocol for this manuscript was not registered in PROSPERO or any other public registry. The study follows standard methodological principles for structured literature reviews; however, at the time of project initiation, protocol registration was not undertaken.

### 2.2. Search Strategy

A comprehensive literature search was performed in PubMed, Web of Science, Scopus, and CAB Abstracts. The search used predefined keyword combinations, including:

“colostrum quality”, “colostrum management”, “passive immunity”, “IgG concentration”, “Brix refractometer”, “serum total protein”, “dairy calves”, “colostrum bank”, “passive transfer failure”, “vaccination during the dry period”.

No restrictions on publication year were applied, allowing inclusion of earlier landmark studies essential for understanding immunoglobulin absorption physiology. Only peer-reviewed articles published in English were eligible.

### 2.3. Eligibility Criteria

#### 2.3.1. Inclusion Criteria

Studies were eligible for inclusion if they met all of the following criteria:

(1) Published in a peer-reviewed scientific journal and written in English.
(2) Conducted in dairy cows and/or dairy calves.
(3) Reported original data on at least one of the following topics:
  - colostrum quality (e.g., IgG concentration, Brix %, serum total protein, bacterial contamination);
  - colostrum collection, hygiene, storage, heat treatment, or feeding protocols;
  - indicators of passive transfer (e.g., serum IgG, STP, Brix) in neonatal calves.
(4) Used clearly described analytical or diagnostic methods (e.g., RID, ELISA, refractometry) or defined management protocols.
(5) Provided extractable quantitative or well-defined qualitative outcomes relevant to colostrum management and PTI.

#### 2.3.2. Exclusion Criteria

Studies were excluded if they:

(1) Focused on species other than cattle;
(2) Investigated only colostrum replacers without comparison to natural colostrum;
(3) Did not report any outcomes related to colostrum quality or PTI (e.g., no IgG, STP, Brix, or health outcomes);
(4) Were narrative reviews, expert opinions, conference abstracts, or case reports without original data;
(5) Lacked sufficient methodological detail (e.g., missing description of IgG measurement, unclear feeding protocol) to allow reliable data extraction.

### 2.4. Study Selection

**Figure 1:**
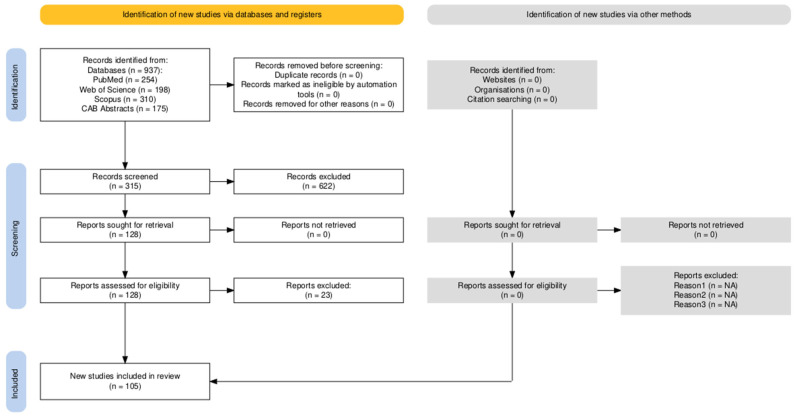
The process of identification, screening, and inclusion of publications is presented in the PRISMA 2020 flow diagram.

All records identified in the database search were imported into a reference management tool, and duplicates were excluded. The selection process followed PRISMA 2020 and is summarized in Figure 1 (PRISMA flow diagram). A total of 937 records were identified across the four databases.

- After removal of duplicates and obviously irrelevant titles, 315 records remained for title and abstract screening.
- Based on this screening, 128 full-text articles were retrieved and assessed for eligibility.
- 23 full-text articles were excluded because they:
  (1) Did not report outcomes relevant to PTI or colostrum management;
  (2) Focused on non-bovine species;
  (3) Evaluated only colostrum replacers without natural colostrum; or;
  (4) Provided insufficient methodological detail.
- 105 studies met all inclusion criteria and were used.

A total of 105 studies were included in the qualitative synthesis. Among them 34 studies contained extractable, methodologically adequate quantitative data required for structured comparative analysis and were therefore included in the evidence synthesis presented in the manuscript. The remaining studies were used only for background narrative support because they lacked extractable outcomes or methodological detail. This approach follows PRISMA standards for transparent, tiered synthesis of heterogeneous evidence.

### 2.5. Data Extraction

Data from each included study were extracted into a standardized template. The following variables were collected:

- Authors, year of publication, and country;
- Study design (experimental, clinical, observational, methodological);
- Target population (dairy cows, colostrum samples, neonatal calves);
- Colostrum-related variables (IgG concentration, Brix %, STP, microbial load; timing and volume of feeding; source of colostrum);
- Analytical and diagnostic methods (RID, ELISA, refractometry, microbiological culture);
- Definitions and thresholds used for PTI or failure of passive transfer (FPT);
- Key outcomes related to colostrum quality, PTI, and calf health.

Where necessary, information was cross-checked within the article (methods, results, tables) to ensure internal consistency.

### 2.6. Risk of Bias Assessment

Risk of bias was assessed for all included studies using adapted critical appraisal checklists appropriate to the study design, based on tools developed by the Joanna Briggs Institute (JBI) and commonly used instruments for observational and diagnostic studies. The following domains were considered:

- Clarity and appropriateness of the study design;
- Selection of animals and representativeness of the study population;
- Validity and reliability of the measurement methods (e.g., IgG assays, Brix/STP devices);
- Control or reporting of potential confounding factors (e.g., calf age at sampling, feeding history, health status);
- Completeness and transparency of reporting (e.g., missing data, selective outcome reporting).

Each study was qualitatively classified as having low, moderate, or high risk of bias. The overall judgment for each study was taken into account in the narrative synthesis and is discussed in the context of the strengths and limitations of the available evidence. Risk of bias results for all included studies are presented in Supplementary Table S1.

### 2.7. Summary of Included Studies

To provide a concise overview of the most influential and representative research, a further subset of key studies has been summarized in Table 1. These studies were selected because they offered the clearest methodological rigor, directly comparable outcome measures, and high relevance to colostrum quality evaluation, colostrum management practices, and PTI assessment in dairy calves. Presenting only this subset enhances interpretability while maintaining transparency about the broader dataset included in the review.

## 3. Results and Discussion

### 3.1. Colostrum Quality Assessment

The immunological value of bovine colostrum is determined primarily by its IgG concentration, with ≥50 g/L widely considered the minimum threshold required for adequate passive transfer of immunity [5]. Substantial variation in IgG levels between cows, parities and farms has been consistently reported, including in large national evaluations [8].

**Laboratory Methods for IgG Determination**

Radial immunodiffusion (RID) remains the reference laboratory method for quantifying colostral IgG. It offers high analytical accuracy and reproducibility but requires specialized equipment and 18–24 h to deliver results, limiting its practicality for immediate decision-making on farms.

**Brix Refractometry**

Brix refractometry is the most widely used on-farm method for rapid assessment of colostrum quality. Numerous studies demonstrate a strong correlation between Brix percentage and IgG concentration measured by RID [10,13,19]. Commonly reported associations include:

- ≈22–23% Brix corresponding to ≥50 g/L IgG;
- <18–20% Brix indicating insufficient IgG content;
- ≥25% Brix indicating high-quality colostrum.

These cut-off values are stable across studies and serve as practical decision thresholds in dairy operations. Both digital and optical refractometers typically require over one minute to obtain an accurate reading, including time for sample stabilization and equipment cleaning.

**Factors Determining Colostrum Quality**

Colostrum IgG concentration is shaped by multiple biological and management factors. Parity is among the most important determinants: multiparous cows generally produce colostrum with higher IgG levels than primiparous cows [10]. Additional influential factors include:

- The interval from calving to first milking, as delays dilute colostrum and reduce IgG concentration;
- The cow’s metabolic and periparturient health status, which may impair colostrogenesis;
- Nutritional status, as inadequate energy or nutrient supply can reduce IgG synthesis.

**Practical Implications**

Routine Brix-based assessment allows fast, standardized and sufficiently accurate decisions regarding whether colostrum should be used for first feeding, stored in a colostrum bank or excluded from use. This contributes to more consistent colostrum-management practices and improved passive transfer outcomes.

Table 2 provides a comparative summary of key methods used to evaluate colostrum quality, outlining their operating principles, recommended cut-off values and diagnostic performance based on validated evidence.

**Critical Analysis**

Although Brix refractometry is widely validated as an effective on-farm screening tool, several limitations warrant consideration. Reported correlations with RID-measured IgG often originate from controlled research conditions, which may not fully reflect variability in breeds, environments and management systems. Sensitivity and specificity values also vary across studies, indicating that herd-specific calibration may be necessary.

RID provides superior analytical accuracy but is impractical for immediate on-farm decisions because of its long processing time. Visual assessment, although still used in some herds, demonstrates consistently poor diagnostic reliability and should not be employed as a standalone method.

Several studies do not fully account for confounding factors such as maternal health, metabolic disorders, udder hygiene or sampling inconsistencies, all of which may influence IgG concentration. The methodological heterogeneity underscores the need for standardized sampling and analytical protocols to strengthen comparability and improve the evidence base for colostrum quality assessment.

### 3.2. Collection, Hygiene, Storage, and Processing of Colostrum

Effective colostrum management relies on rigorous control of collection hygiene, rapid cooling, appropriate storage conditions, controlled thawing, and validated heat-treatment procedures. These steps are critical because bacterial contamination and improper storage can reduce immunoglobulin G (IgG) concentration, impair absorption, and increase the risk of failure of FPT. A large-scale Czech survey reported substantial microbial contamination in harvested colostrum, highlighting the need for standardized hygiene and handling practices [20].

**Colostrum Collection and Milking Hygiene**

Microbial contamination is one of the leading causes of reduced IgG availability and PTI. In a dataset of >300 samples from commercial dairy farms, only 28.4% met the recommended standard of <100,000 cfu/mL total plate count (TPC), while 88.2% met the <10,000 cfu/mL coliform threshold [20]. These findings align with the microbial safety guidelines summarized by Denholm [21], who emphasized the importance of hygienic collection to limit environmental contamination.

Key hygienic procedures include:

- Milking as soon as possible after calving—ideally within 2 h—to limit bacterial proliferation;
- Thorough cleaning and drying of teats to reduce skin, bedding and faecal contamination;
- Use of sanitized containers, tubes and milking equipment to prevent cross-contamination;
- Immediate cooling of colostrum prior to storage or feeding.

Multiparous cows generally produce colostrum with higher IgG and lower variability, making them suitable donors for farm colostrum banks.

**Cooling and Refrigerated Storage**

Bacteria multiply rapidly when colostrum remains at ambient temperature. Denholm [21] notes that microbial populations may double every 20–30 min at room temperature, underscoring the need for prompt cooling.

Evidence shows that:

- Storage at 22 °C for 48 h leads to substantial increases in TPC, decreases in pH and lower serum IgG in calves fed this colostrum [22];
- Refrigeration at 4 °C for 24–48 h preserves IgG concentration, although bacterial counts gradually increase over time [22,23].

Refrigerated colostrum should be used within 48 h unless frozen for longer-term storage.

**Freezing and Thawing**

Freezing is an essential method for long-term colostrum preservation, especially for building high-quality colostrum banks.

A recent controlled study [24] demonstrated that:

- Freezing at −20 °C for up to 1 year leads to a modest ~8% decline in IgG by 32–48 weeks.
- Brix values and insulin concentrations also decline over extended storage.
- Coliform counts decrease during frozen storage, improving hygienic quality.

Based on these data, optimal quality retention is achieved with frozen storage ≤ 32 weeks.

Thawing guidelines:

- Thaw gently in a warm-water bath ≤50 °C, avoiding high temperatures.
- Temperatures >60 °C cause irreversible IgG denaturation and excessive viscosity [21].

To ensure uniform thawing, colostrum should be agitated gently throughout warming.

**Heat Treatment (Pasteurization)**

Pasteurization of colostrum can effectively reduce pathogenic and indicator bacteria while preserving IgG functionality if performed correctly.

The most validated method is:

- Low-temperature, long-time (LTLT) heat treatment: 60 °C for 60 min.

Scientific evidence demonstrates that this protocol:

- Reduces coliforms and TPC by ~2 log10;
- Maintains colostrum viscosity within acceptable ranges [13];
- Preserves IgG concentration sufficiently for effective passive transfer;
- Improves serum IgG and calf health outcomes after feeding heat-treated colostrum [14].

Higher temperatures (≥63–72 °C) cause significant IgG degradation and viscosity changes, and therefore are not suitable for bovine colostrum processing.

**Practical Recommendations and Integration at Farm Level**

Based on validated scientific evidence, optimal colostrum handling should follow these key steps:

- Early milking (≤2 h) to capture maximal IgG concentration.
- Strict hygiene of udder and equipment during colostrum harvest.
- Rapid cooling to ≤4 °C immediately after collection.
- Refrigerated storage ≤48 h, with monitoring of temperature and labeling.
- Frozen storage at −20 °C, ideally not exceeding 32 weeks.
- Thawing at ≤50 °C with continuous agitation.
- Optional LTLT pasteurization at 60 °C for 60 min for microbial control.

This integrated approach reduces microbial contamination, preserves IgG, and enhances the likelihood of successful passive transfer. Table 3 presents recommended practices for colostrum collection, hygiene, storage and processing.

**Critical Analysis**

Although the body of literature on colostrum handling is substantial, several limitations should be considered when interpreting the available evidence. Many studies originate from high-input dairy systems, which may restrict the generalizability of specific microbial thresholds or storage recommendations to different production environments [20,21]. Research on colostrum storage conditions often relies on relatively small experimental cohorts, limiting the strength of conclusions concerning the effects of temperature and duration on IgG stability and bacterial proliferation [22,23]. Similarly, studies evaluating heat treatment demonstrate considerable heterogeneity in equipment type, batch size, temperature control, and farm-level compliance, which contributes to variable reports of IgG preservation and viscosity changes [13,14]. Emerging preservation techniques, including alternative pasteurization technologies, remain insufficiently validated under field conditions and therefore cannot yet be recommended for routine use. Despite these limitations, the evidence consistently indicates that early hygienic collection, rapid cooling, controlled short- or long-term storage, and validated heat treatment protocols form the foundation of effective colostrum management and significantly enhance the likelihood of successful passive transfer in neonatal calves.

### 3.3. Timing, Volume, and Frequency of Colostrum Feeding

Achieving adequate PTI in newborn calves depends on timely intake of sufficient high-quality colostrum delivered through an appropriate feeding method. Intestinal permeability to IgG declines rapidly after birth; delaying colostrum provision beyond the first hours postpartum markedly reduces the apparent efficiency of absorption and can negatively affect early gut microbial colonization [25]. Feeding protocols must therefore optimize (1) the timing of the first feeding, (2) the total IgG mass delivered and (3) the method used to administer colostrum.

**Timing of First Colostrum Feeding**

Newborn calves should receive their first colostrum feeding within 1–2 h postpartum, when IgG absorption efficiency is at its peak and the risk of failed passive transfer is lowest [5,26]. Absorption begins to decline after approximately two hours, and by 6–8 h postpartum intestinal uptake capacity may decrease by 50% [25]. Feeding should not be delayed beyond 6 h, as repeatedly demonstrated in classical and contemporary reviews [1]. Controlled trials consistently show that administering colostrum within the first 1–2 h results in the highest serum IgG concentrations and minimizes the likelihood of FPT [5,26].

**Volume and IgG Mass Required**

To achieve successful passive transfer, calves must ingest an adequate mass of IgG. Consistent findings indicate the need for at least 150–200 g of IgG at first feeding [5,23]. This is typically achieved by:

- 3–4 L of high-quality colostrum (≥50 g/L IgG);
- 10% of birth bodyweight at the first feeding.

Offering a second feeding of 2 L of high-quality colostrum or transition milk within the first 12 h further improves serum IgG concentration and health outcomes [5,15].

Colostrum source also plays a significant role: colostrum from multiparous cows generally provides higher IgG concentrations and superior passive transfer compared with that from primiparous cows [27].

**Feeding Methods**

Feeding method influences the likelihood that calves ingest the recommended volume of colostrum. Bottle feeding is preferred when the calf drinks voluntarily, as it activates the esophageal groove reflex and supports physiological digestion. However, many newborn calves fail to drink sufficient volumes in the first hours of life [4]. Evidence shows that:

- Bottle feeding is effective when calves readily consume ≥3 L.
- Oroesophageal tube feeding is safe and results in equivalent serum IgG concentrations when high-quality colostrum is used [16,28].
- Tube feeding should be used without delay when the calf fails to voluntarily ingest ≥3 L in the first 2 h [29].

These findings support a decision-based approach, where bottle feeding is attempted first, followed by timely tube feeding to ensure adequate IgG intake. Table 4 presents evidence-based recommendations for timing, volume, and methods of colostrum feeding in dairy calves [30].

**Critical Analysis**

Although strong evidence-based data supports early and adequate colostrum feeding, substantial variation persists among dairy farms in the implementation of feeding protocols. A major limitation in practice is the dependence on calf vigor; bottle feeding is effective only when calves voluntarily consume sufficient volume. Overreliance on voluntary intake contributes to underfeeding, one of the primary causes of FPT. Conversely, although tube feeding circumvents this issue, it bypasses the esophageal groove reflex. Nonetheless, multiple controlled studies confirm that when performed correctly, tube feeding does not reduce serum IgG concentrations and should be used promptly when calves fail to drink.

Recent research emphasizes that parity-related differences in colostrum quality must be integrated into feeding protocols; ignoring this factor may lead to suboptimal IgG delivery when colostrum from primiparous cows is used. Further research is needed to clarify the interaction between colostrum composition, bacterial load, feeding frequency, and gut microbiome development.

The evidence strongly supports a structured, proactive feeding strategy—combining early administration, adequate IgG mass, and an adaptive approach to feeding method—to reduce FPT and optimize neonatal calf immunity.

### 3.4. Assessment of Calf Immunity via Serum IgG

Evaluating PTI in neonatal calves is essential for assessing the effectiveness of colostrum management and identifying animals at increased risk for morbidity, reduced growth and mortality. Since calves are born agammaglobulinemic and rely entirely on maternally derived immunoglobulin G (IgG), serum IgG concentration measured during the first 24–48 h postpartum provides a direct and quantitative indicator of PTI success [1]. Numerous studies confirm that calves with inadequate IgG concentrations have an elevated likelihood of diarrheal and respiratory disease, greater need for antimicrobial treatment and poorer long-term performance [27,29].

**Reference Laboratory Method: Radial Immunodiffusion (RID)**

RID is the gold-standard laboratory assay for measuring serum IgG. It offers excellent analytical specificity and accuracy, but requires 18–24 h to complete, trained personnel and dedicated laboratory facilities. As a result, RID is used primarily for validation studies and research, while faster on-farm methods are preferred for routine monitoring [1].

**Serum Total Protein (STP) by Refractometry**

STP is one of the most widely implemented on-farm diagnostic indicators of passive transfer. It correlates strongly with serum IgG in clinically normal, adequately hydrated calves. Classical work indicates that an STP concentration of approximately 5.2 g/dL corresponds to serum IgG ≈ 10 g/L, making this value an accepted operational threshold for defining adequate PTI [1]. More recent evaluations show that both optical and digital refractometers provide reliable performance when used consistently [31]. Although STP can be influenced by dehydration, inflammation or hemoconcentration, it remains a practical and cost-effective screening tool for herd-level monitoring [17].

**Serum Brix Percentage**

Brix refractometry, traditionally used for measuring colostrum solids, has also been validated as an on-farm method for estimating serum IgG. Deelen et al. [18] identified a Brix value of approximately 8.4% as the optimal threshold for diagnosing calves with IgG concentrations below 10 g/L. Subsequent studies confirm that serum Brix values between 8.1% and 8.5% reliably distinguish adequate from inadequate PTI [17,18]. Because Brix refractometers are durable, inexpensive and already common on dairy farms for colostrum testing, their application for serum evaluation offers significant practical advantages.

**Classification Thresholds for Passive Transfer**

Based on comprehensive expert reviews and modern field studies, PTI is classified as follows [5,26]:

- Adequate passive transfer: ≥10 g/L IgG;
- Partial failure: 5–9.9 g/L IgG;
- FPT: <5 g/L IgG.

These thresholds are compatible with RID-derived IgG values and align with epidemiological herd benchmarks [17]. Table 5 summarizes validated diagnostic methods for evaluating passive immunity in neonatal calves.

**Critical Analysis**

Although RID remains the most accurate technique for quantifying serum IgG, its operational constraints limit its routine on-farm application. Refractometry-based approaches, particularly STP and Brix measurements, provide rapid, economical and sufficiently robust alternatives for large-scale herd monitoring. Their diagnostic performance can be affected by calf hydration status, sample quality and operator technique. Standardized sampling protocols and consistent device calibration are essential for reliable field use.

Recent studies emphasize that no single cutoff is universally applicable across all herds, as biological variability, management practices and disease pressure influence IgG dynamics [17]. Integrating both STP and serum Brix measurements into neonatal health programs can improve diagnostic accuracy and enable more nuanced herd-specific assessment of passive transfer status. Reliable evaluation of serum IgG, combined with colostrum quality assessment and timely first feeding, forms a critical triad for reducing FPT and supporting optimal calf health and productivity.

**Practical Recommendations**

Based on the evidence synthesized in this systematic review, several practical recommendations can be outlined to optimize colostrum management and ensure adequate PTI in dairy calves. These recommendations are drawn from validated studies investigating colostrum quality, feeding protocols, IgG absorption kinetics and post-feeding immunological assessment [1,5,26,27,29,30,31].

**(1)**  **Implement Structured Decision-Making Protocols**

Colostrum management should rely on predefined procedures rather than ad hoc decisions made at the time of calving. Farms benefit from clearly documented steps outlining who is responsible for collection, testing, feeding and monitoring. Standardization reduces variation in practice and helps ensure that essential actions—such as timely feeding and quality assessment—are performed consistently across all staff members and work shifts [5,29,30].

**(2)**  **Integrate Quality Assessment Into Routine Colostrum Handling**

Colostrum testing should be embedded in daily workflows so that quality evaluation becomes an automatic part of handling rather than an optional activity. Farms should designate specific points in the process (e.g., immediately after milking) at which quality is assessed using validated tools such as refractometry. This approach supports better allocation decisions and helps maintain the reliability of colostrum banks [17,18,30].

**(3)**  **Align Feeding Strategies With Calf Condition and Farm Logistics**

Protocols should provide flexibility to adapt feeding methods and volumes based on calf vigor, staffing capacity and the availability of high-quality colostrum. Clear criteria defining when to transition from bottle feeding to assisted feeding (e.g., tube administration) improve consistency and ensure that calves receive adequate IgG even when voluntary intake is limited [16,28,29].

**(4)**  **Adopt a Comprehensive Hygiene and Storage Framework**

Hygiene, cooling, storage and thawing should be treated as interconnected components of a single management system rather than isolated steps. Farms should maintain written hygiene protocols, monitor microbial contamination trends and routinely review whether cooling and storage capacities align with herd size and calving intensity [20,21,22]. Integrating these elements into a unified framework minimizes bacterial proliferation and supports more predictable IgG absorption outcomes.

**(5)**  **Use PTI Monitoring Data to Drive Continuous Improvement**

Routine measurement of serum IgG indicators allows farms to evaluate the effectiveness of their colostrum protocols objectively. Aggregated herd data—such as the distribution of STP or Brix values in newborn calves—can highlight bottlenecks, reveal seasonal patterns and identify where procedural adjustments are needed [1,17,18]. Farms with consistently high rates of partial or failed PTI may require targeted interventions such as adjustments in staff training or revisions to storage capacity, feeding timing or colostrum sourcing [26].

**(6)**  **Prioritize Staff Training and Skills Consolidation**

High-quality colostrum management depends on the competence and confidence of personnel involved in calving and newborn care. Structured training programs should be implemented regularly, ensuring that employees understand not only the procedural steps but also the rationale behind them. Emphasis on correct equipment use, hygiene, and calf evaluation promotes more uniform execution of protocols and reduces reliance on individual experience [27,29,30].

**(7)**  **Adapt Protocols to Herd-Specific Risk Profiles**

Farms differ in herd demographics, disease pressure, climate, management intensity and calving distribution. A one-size-fits-all protocol is unlikely to be fully effective across diverse production systems. Management strategies should therefore be evaluated periodically and adapted to reflect local constraints and risk factors, such as high rates of dystocia, seasonal weather extremes or frequent delays between calving and first feeding [17,26]. Tailoring these adjustments ensures that general scientific recommendations translate effectively into farm-specific practice.

This systematic review has several limitations that should be considered when interpreting its findings. Although the review followed the core elements of the PRISMA 2020 framework, the protocol was not prospectively registered in a public database, which may increase the potential for selection or reporting bias. The included studies also demonstrated substantial heterogeneity in design, sample size, management conditions, analytical techniques, and definitions of passive transfer, which limited the ability to perform standardized comparisons or quantitative synthesis.

Differences in laboratory methodologies used to quantify IgG, such as RID, ELISA, or refractometry-based estimations—as well as inconsistent reporting of bacterial contamination thresholds, colostrum handling procedures, or precise feeding protocols—may have affected the accuracy and comparability of extracted data across studies. Additionally, variation in full-text availability between databases and the exclusion of non-English publications may have introduced risk of publication bias.

Although an adapted risk-of-bias appraisal was applied using JBI- and QUADAS-2-based domains, many included studies were observational or field-based and often lacked complete methodological reporting. As a result, some uncertainty remains regarding the strength and generalizability of specific findings. Furthermore, while recent research was incorporated, rapidly emerging technologies—such as automated colostrum feeders, digital monitoring platforms, and precision-based IgG evaluation tools—are not yet widely represented in the available literature and may influence future recommendations.

Despite these limitations, the review synthesizes the current evidence in a structured and transparent manner and highlights consistently supported practices that are essential for improving passive immunity transfer and early-life health outcomes in dairy calves.

## 4. Conclusions

Effective colostrum management is essential for establishing early immunity and supporting the long-term health and productivity of dairy calves. The evidence reviewed in this work demonstrates that colostrum quality, hygienic handling, timely administration and appropriate monitoring should be viewed as interconnected components of a single management system rather than independent practices. When these elements function coherently, they substantially improve the likelihood of achieving successful passive transfer.

The literature consistently highlights that calves receiving adequate passive immunity experience lower morbidity, improved growth and better overall performance throughout rearing. Nevertheless, considerable variability persists among farms in how colostrum is collected, stored, evaluated and fed, underscoring the importance of structured protocols and continuous quality control. Integrating routine colostrum assessment with regular monitoring of serum IgG indicators provides farms with practical feedback that can guide protocol refinement and help identify areas requiring targeted intervention.

No single measure alone is sufficient to ensure reliable outcomes. Instead, successful passive transfer depends on the coordinated application of evidence-based practices carried out by trained personnel and adapted to the specific conditions of each herd. Adoption of such integrated, consistently applied strategies strengthens neonatal immunity, reduces early-life disease burden and contributes meaningfully to the long-term sustainability and productivity of dairy operations.

## Figures and Tables

**Table 1:** Summary of Key Studies Included in the Systematic Review.

Country	Study Design	Sample Size	Study Focus	Methods	Key Findings	Authors (Year)
USA	Observational	827 colostrum samples	Colostrum IgG variability	RID, bacterial culture	30% < 50 g/L IgG; large farm-level variation	[8]
Canada	Method validation	200 calves	Brix/STP correlation	Brix, STP, RID	High correlation with serum IgG	[10]
USA	Method validation	183 samples	Brix validation	Brix, RID	21% Brix = ≥50 g/L IgG	[12]
USA	Field study	Colostrum pools	Microbial load	RID, culture	Heat treatment reduces pathogens significantly	[13]
USA	Experimental	Colostrum	Heat treatment	60 °C LTLT	Major bacterial reduction; minimal IgG loss	[14]
Ireland	Experimental	99 calves	Volume and IgG mass	ELISA	8.5% BW improves IgG absorption	[15]
USA	Clinical	Calves	Feeding method	Tube vs. bottle	Similar serum IgG with both methods	[16]
Australia	Observational	Calves	Colostrum source	RID	Multiparous cows produce higher IgG colostrum	[17]
Canada	Population study	>500 calves	STP and Brix thresholds	STP, Brix, RID	Validated STP/Brix thresholds	[17]
USA	Method validation	Calves	Serum Brix	Brix, RID	8.4% optimal cut-off for FPT detection	[18]

Abbreviations: IgG—immunoglobulin G; RID—radial immunodiffusion; STP—serum total protein; BW—body weight; LTLT—low-temperature long-time pasteurization.

**Table 2:** Methods Used for Colostrum Quality Assessment and Their Diagnostic Characteristics.

Method	Principle	Recommended Cut-Off Values	Diagnostic Characteristics	Main Reference
Radial immunodiffusion (RID)	Laboratory quantification of IgG via antigen–antibody precipitation	≥50 g/L IgG	Reference method; high accuracy; slow turnaround time	[5]
Optical Brix refractometry	Measurement of total soluble solids correlating with IgG	22% Brix ≈ 50 g/L IgG	Correlation r ≈ 0.80–0.86 vs. RID	[19]
Digital Brix refractometry	High-precision Brix measurement with enhanced repeatability	22–23% Brix	Sensitivity 80–90%; specificity 70–90%	[10]
Visual assessment	Evaluation based on color and viscosity	No valid cut-off	Low accuracy; not recommended	[12]
Indirect physical indicators (density, specific gravity)	Estimation of physical properties	No standardized cut-off	High variability; limited reliability	[5]
Infrared spectroscopy	Analysis of compositional parameters (fat, protein)	No IgG-specific cut-off	Useful for composition; cannot replace IgG measurement	[8]

Abbreviations: IgG—immunoglobulin G, RID—radial immunodiffusion, PTI—passive transfer of immunity, FPT—failure of passive transfer, Brix—percentage of total soluble solids.

**Table 3:** Recommended Practices for Colostrum Collection, Hygiene, Storage, and Processing.

Step	Recommended Parameters	Practical Importance	References
Hygienic collection	Clean teats; sanitized equipment	Reduces microbial contamination	[20,21]
Microbial thresholds	TPC < 100,000 cfu/mL; coliforms < 10,000 cfu/mL	Defines acceptable colostrum for feeding	[20,21]
Cooling	≤4 °C immediately after collection	Minimizes bacterial growth	[23]
Refrigerated storage	4 °C for ≤48 h	Maintains IgG; limits microbial proliferation	[22,23]
Warm storage (avoid)	13–22 °C (not recommended)	Leads to exponential bacterial growth and reduced serum IgG	[22]
Freezing	−20 °C; ≤32 weeks	Good IgG preservation; reduced coliforms	[24]
Thawing	≤50 °C in warm-water bath	Prevents IgG denaturation	[21]
Heat treatment	60 °C for 60 min (LTLT)	Reduces bacteria; preserves IgG	[13,14]

Abbreviations: TPC—total plate count, cfu—colony-forming units, IgG—immunoglobulin G, LTLT—low-temperature, long-time pasteurization (60 °C for 60 min).

**Table 4:** Evidence-based recommendations for timing, volume, and methods of colostrum feeding in dairy calves.

Aspect	Evidence-Based Recommendation	Supporting Studies
Timing of first feeding	Within 1–2 h after birth	[5,25]
Maximum delay	Not later than 6 h postpartum	[1]
IgG mass required	≥150–200 g IgG at first feeding	[5,15]
Volume	3–4 L (or 10% BW) of high-quality colostrum	[15]
Second feeding	2 L within 12 h	[5]
Preferred method	Bottle feeding when calf drinks voluntarily	[16,28]
When to tube-feed	If calf fails to ingest ≥3 L within 2 h	[29]
Colostrum source effect	Multiparous cows produce higher-IgG colostrum	[27]

Abbreviations: IgG—immunoglobulin G, PTI—passive transfer of immunity, FPT—failure of passive transfer, AEA—apparent efficiency of absorption, BW—birth bodyweight.

**Table 5:** Validated diagnostic methods for assessing passive immunity in neonatal dairy calves.

Method	Measurement Principle	Threshold for Adequate PTI	Advantages	Limitations	Key References
RID (reference)	Antigen–antibody diffusion to quantify IgG	IgG ≥ 10 g/L	Gold standard; highest accuracy	Slow; laboratory-dependent	[1]
Serum Total Protein (STP)	Optical or digital refractometry	5.0–5.5 g/dL	Rapid; low cost; useful for herd monitoring	Affected by hydration and systemic disease	[17,31]
Serum Brix (%)	Light refraction (total soluble solids)	8.1–8.5% Brix (optimal ≈ 8.4%)	Practical; consistent; uses same device as colostrum testing	Slightly less precise than RID	[17,18]

Abbreviations: IgG—immunoglobulin G; PTI—passive transfer of immunity; FPT—failure of passive transfer; RID—radial immunodiffusion; STP—serum total protein.

## Data Availability

No new data were created or analyzed in this study. Data sharing is not applicable to this article.

## Supplementary Materials

The following supporting information can be downloaded at: https://www.mdpi.com/article/10.3390/vetsci12121205/s1, Table S1: Risk of bias assessment.

## References

[1] Weaver D.M., Tyler J.W., VanMetre D.C., Hostetler D.E., Barrington G.M. (2000). Passive Transfer of Colostral Immunoglobulins in. J. Vet. Intern. Med..

[2] Calves Larson B.L., Heary H.L., Devery J.E. (1980). Immunoglobulin Production and Transport by the Mammary Gland. J. Dairy Sci..

[3] McGuirk S.M., Collins M. (2004). Managing the Production, Storage, and Delivery of Colostrum. Vet. Clin. N. Am. Food Anim. Pract..

[4] Godden S.M. (2008). Colostrum Management for Dairy Calves. Vet. Clin. N. Am. Food Anim. Pract..

[5] Godden S.M., Lombard J.E., Woolums A.R. (2019). Colostrum Management for Dairy Calves. Vet. Clin. N. Am. Food Anim. Pract..

[6] Stott G.H., Marx D.B., Menefee B.E., Nightengale G.T. (1979). Colostral Immunoglobulin Transfer in Calves. I. Period of Absorption. J. Dairy Sci..

[7] Stott G.H., Marx D.B., Menefee B.E., Nightengale G.T. (1979). Colostral Immunoglobulin Transfer in Calves. II. The Rate of Absorption. J. Dairy Sci..

[8] Morrill K.M., Conrad E., Lago A., Campbell J., Quigley J., Tyler H. (2012). Nationwide Evaluation of Quality and Composition of Colostrum on Dairy Farms in the United States. J. Dairy Sci..

[9] Lombard J.E., Urie N.J., Garry F.B., Godden S.M., Quigley J.D., Earleywine T.J., McGuirk S.M., Moore D.A., Branan M.A., Chamorro M.F. (2020). Consensus Recommendations on Calf- and Herd-Level Passive Immunity in Dairy Calves in the United States. J. Dairy Sci..

[10] Elsohaby I., McClure J.T., Cameron M., Heider L.C., Keefe G.P. (2017). Rapid Assessment of Bovine Colostrum Quality: How Reliable Are Transmission Infrared Spectroscopy and Digital and Optical Refractometers?. J. Dairy Sci..

[11] Westhoff T.A., Borchardt S., Mann S. (2024). Invited Review: Nutritional and Management Factors That Influence Colostrum Production and Composition in Dairy Cows. J. Dairy Sci..

[12] Bartier A.L., Windeyer M.C., Doepel L. (2015). Evaluation of On-Farm Tools for Colostrum Quality Measurement. J. Dairy Sci..

[13] Godden S., McMartin S., Feirtag J., Stabel J., Bey R., Goyal S., Metzger L., Fetrow J., Wells S., Chester-Jones H. (2006). Heat Treatment of Bovine Colostrum II: Effects of Heating Duration on Pathogen Viability and Immunoglobulin G. J. Dairy Sci..

[14] Donahue M., Godden S.M., Bey R., Wells S., Oakes J.M., Sreevatsan S., Stabel J., Fetrow J. (2012). Heat Treatment of Colostrum on Commercial Dairy Farms Decreases Colostrum Microbial Counts while Maintaining Immunoglobulin G Concentrations. J. Dairy Sci..

[15] Conneely M., Berry D.P., Murphy J.P., Lorenz I., Doherty M.L., Kennedy E. (2014). Effect of Feeding Colostrum at Different Volumes and Subsequent Number of Transition Milk Feeds on Serum IgG Concentration and Health Status of Dairy Calves. J. Dairy Sci..

[16] Chigerwe M., Coons D.M., Hagey J.V. (2012). Comparison of Colostrum Feeding by Nipple Bottle versus Oroesophageal Tubing in Holstein Dairy Bull Calves. J. Am. Vet. Med. Assoc..

[17] Sutter F., Gómez D.E., Arroyo L.G., Socha M., Renaud D.L. (2020). Evaluation of Analytical Methods to Assess Failure of Transfer of Passive Immunity in Dairy Calves. J. Dairy Sci..

[18] Deelen S.M., Ollivett T.L., Haines D.M., Leslie K.E. (2014). Evaluation of a Brix Refractometer to Estimate Serum Immunoglobulin G Concentration in Neonatal Dairy Calves. J. Dairy Sci..

[19] Bielmann V., Gillan J., Perkins N.R., Skidmore A.L., Godden S., Leslie K.E. (2010). An Evaluation of Brix Refractometry Instruments for Measurement of Colostrum Quality in Dairy Cattle. J. Dairy Sci..

[20] Slosarkova S., Pechova A., Stanek S., Fleischer P., Zouharova M., Nejedla E. (2021). Microbial Contamination of Harvested Colostrum on Czech Dairy Farms. J. Dairy Sci..

[21] Denholm K. (2022). A Review of Bovine Colostrum Preservation Techniques. J. Dairy Res..

[22] Cummins C., Berry D.P., Murphy J.P., Lorenz I., Kennedy E. (2017). The Effect of Colostrum Storage Conditions on Dairy Heifer Calf Serum Immunoglobulin G Concentration and Preweaning Health and Growth Rate. J. Dairy Sci..

[23] Cummins C., Lorenz I., Kennedy E. (2016). The Effect of Storage Conditions over Time on Bovine Colostral Immunoglobulin G Concentration, Bacteria, and pH. J. Dairy Sci..

[24] Westhoff T.A., Mann S. (2025). Effect of Frozen Storage of Bovine Colostrum for up to 1 Year on Concentrations of Immunoglobulins and Insulin as Well as Bacterial Counts. JDS Commun..

[25] Fischer A.J., Song Y., Haines D.M., Guan L.L., Steele M.A. (2018). Effect of Delaying Colostrum Feeding on Passive Transfer and Intestinal Bacterial Colonization in Neonatal Male Holstein Calves. J. Dairy Sci..

[26] Robbers L., Jorritsma R., Nielen M., Koets A. (2021). A Scoping Review of On-Farm Colostrum Management Practices for Optimal Transfer of Immunity in Dairy Calves. Front. Vet. Sci..

[27] Hue D.T., Skirving R., Chen T., Williams J.L., Bottema C.D.K., Petrovski K. (2021). Colostrum Source and Passive Immunity Transfer in Dairy Bull Calves. J. Dairy Sci..

[28] Desjardins-Morrissette M., van Niekerk J.K., Haines D., Sugino T., Oba M., Steele M.A. (2018). The Effect of Tube versus Bottle Feeding Colostrum on Immunoglobulin G Absorption, Abomasal Emptying, and Plasma Hormone Concentrations in Newborn Calves. J. Dairy Sci..

[29] Godden S.M., Haines D.M., Konkol K., Peterson J. (2009). Improving Passive Transfer of Immunoglobulins in Calves. II: Interaction between Feeding Method and Volume of Colostrum Fed. J. Dairy Sci..

[30] Tyler J.W., Hancock D.D., Parish S.M., Rea D.E., Besser T.E., Sanders S.G., Wilson L.K. (1996). Evaluation of Three Assays for Failure of Passive Transfer in Calves. J. Vet. Intern. Med..

[31] Elsohaby I., McClure J.T., Keefe G.P. (2015). Evaluation of Digital and Optical Refractometers for Assessing Failure of Transfer of Passive Immunity in Dairy Calves. J. Vet. Intern. Med..

