# Supplementary material for: Good Veterinary Practices for Managing the Colostral Period in Dairy Calves to Improve Their Immune Competence—A Systematic Review
Source: Vet Sci. 2025 Dec 16;12(12):1205. doi: 10.3390/vetsci12121205 (PMC12737328; doi:10.3390/vetsci12121205)
Supplement: Supplementary file 1 [file vetsci-12-01205-s001.zip › vetsci-4010036-Supplementary Materials.pdf]

Table S1. Risk of bias assessment of the 34 studies included in the systematic review, evaluated using adapted Joanna Briggs Institute (JBI) critical appraisal tools for observational studies and QUADAS-2 for diagnostic-accuracy studies. Studies were judged as low, moderate, or high risk of bias across five domains: study design clarity, population selection, measurement validity, control of confounding, and outcome reporting.

Study ID	First Author, Year	Study Type	Tool Used	Design Clarity	Population Selection	Measurement Validity	Confounding Control	Reporting Transparency	Overall Risk of Bias
1	Weaver, 2000	Observational	JBI	Low	Low	Low	Moderate	Low	Low
2	Larson, 1980	Experimental	JBI	Moderate	Moderate	Low	Moderate	Moderate	Moderate
3	McGuirk, 2004	Review/Guideline	– (not assessed)	–	–	–	–	–	Not applicable
4	Godden, 2008	Review	–	–	–	–	–	–	Not applicable
5	Godden, 2019	Review	–	–	–	–	–	–	Not applicable
6	Stott, 1979a	Experimental	JBI	Low	Low	Moderate	Moderate	Moderate	Moderate
7	Stott, 1979b	Experimental	JBI	Low	Low	Moderate	Moderate	Moderate	Moderate
8	Morrill, 2012	Observational	JBI	Low	Low	Low	Moderate	Low	Low
9	Lombard, 2020	Observational (Consensus)	JBI	Low	Low	Low	Moderate	Low	Low
10	Elsohaby, 2017	Diagnostic accuracy	QUADAS-2	Low	Low	Low	Low	Low	Low
11	Westhoff, 2024	Review	–	–	–	–	–	–	Not applicable
12	Bielmann, 2010	Diagnostic	QUADAS-2	Low	Moderate	Low	Moderate	Low	Moderate
13	Bartier, 2015	Observational	JBI	Low	Low	Low	Low	Low	Low
14	Slosarkova, 2021	Observational	JBI	Low	Low	Moderate	Moderate	Low	Moderate
15	Denholm, 2022	Review	–	–	–	–	–	–	Not applicable
16	Cummins, 2016	Experimental	JBI	Low	Low	Low	Moderate	Low	Low
17	Cummins, 2017	Experimental	JBI	Low	Low	Low	Moderate	Low	Low
18	Westhoff, 2025	Experimental	JBI	Low	Low	Low	Moderate	Low	Low
19	Donahue, 2012	Experimental	JBI	Low	Low	Low	Moderate	Low	Low
20	Godden, 2006	Experimental	JBI	Low	Low	Low	Moderate	Low	Low
21	Fischer, 2018	Experimental	JBI	Low	Low	Low	Low	Low	Low
22	Robbers, 2021	Review	–	–	–	–	–	–	Not applicable

Study ID	First Author, Year	Study Type	Tool Used	Design Clarity	Population Selection	Measurement Validity	Confounding Control	Reporting Transparency	Overall Risk of Bias
23	Conneely, 2014	Observational	JBI	Low	Low	Low	Moderate	Low	Low
24	Hue, 2021	Observational	JBI	Low	Low	Low	Moderate	Low	Low
25	Chigerwe, 2012	Experimental	JBI	Low	Low	Low	Moderate	Low	Low
26	Desjardins-Morrissette, 2018	Experimental	JBI	Low	Low	Low	Moderate	Low	Low
27	Godden, 2009	Experimental	JBI	Low	Low	Low	Moderate	Low	Low
28	Tyler, 1996	Diagnostic	QUADAS-2	Moderate	Moderate	Low	Low	Moderate	Moderate
29	Weaver, 2000 (duplicate citation in manuscript sections)	Observational	JBI	Low	Low	Low	Moderate	Low	Low
30	Hue, 2021	Observational	JBI	Low	Low	Low	Moderate	Low	Low
31	Deelen, 2014	Diagnostic	QUADAS-2	Low	Low	Low	Low	Low	Low
32	Elsohaby, 2015	Diagnostic	QUADAS-2	Low	Low	Low	Low	Low	Low
33	Sutter, 2020	Diagnostic	QUADAS-2	Low	Low	Low	Low	Low	Low
34	Robbers, 2021 (scoping)	Review	–	–	–	–	–	–	Not applicable

Legend: Low = high methodological validity, negligible risk of systematic error.

Moderate = some methodological limitations that may influence interpretation.

High = substantial limitations that significantly reduce reliability.

Review studies were not formally assessed for risk of bias, consistent with PRISMA guidance for narrative and scoping reviews.
